# Transactions of the Illinois State Medical Society for 1854

**Published:** 1854-11

**Authors:** 


### BOOK NOTICES.

*Transactions of the Illinois State Medical Society for* 1854. Chicago: J. F. BALLANTYNE, Printer.

This is the title of a volume of 112 pages, printed in very good style, and containing the proceedings of the Illinois State Medical Society, held in June last, together with the Annual Address of the President, Prof. Brainard; a paper on the treatment of deformities of the lower extremities, by Dr. E. S. Cooper; the Prize Essay on the difference between Stimulants and Tonics, by Dr. H. Parker, of Chicago; and the list of Members of the Societ The proceedings of the Annual Meeting, and the Address on the Treatment of Poisoned Wounds, by Prof. D. Brainard, have already been published in former numbers of this Journal. The paper of Dr. Cooper occupies sixteen pages of the volume, and bears the following title, viz.: "Walking rendered the Primary Element in the Cure of I eformities of the Lower Extremities; its early Adaptation to White Swelling and Coxalgia, with Apparatus for carrying out the designs of the same."

In reference to the construction and application of instruments or machines to aid in the removal of deformities, Dr. Cooper gives the following rules, viz.:

"1st. They should press equally upon the largest possible amount of surface over which they may be applied.

2d. To change the points of pressure, whenever required, to favor a tender part, without disturbance to the treatment.

3d. To cause no material interruption to the circulation of the blood.

4th. To permit the free exercise of the limb when not prevented by other causes.

5th. To adapt themselves consistently to the changes of the limb during the process of cure.

6th. In the lower extremities, to facilitate walking while the cure is in progress, and, in most instances, render it the active agent in removing the deformity."

In attempting to apply these rules practically, Dr. Cooper treats more particularly of "club-foot," false anchylosis of the *knee* joint, and scrofulous disease both of the knee and hip. In reference to the treatment of all these diseases the author's views are somewhat novel, and worthy of a careful perusal. But we could not give a fair representation of them without quoting his paper entire, or writing one of our own of nearly equal length. Hence those who desire further knowledge on the subject must refer directly to the paper in the *Transactions*.

The next and last paper contained in the volume before us is the Prize Essay on Stimulants and Tonics, by Henry Parker, M.D., of Chicago. It occupies 54 pages, and is written in good style. The author founds his essay on the following distinct propositions, viz.:

"1st. That all living organized matter is endowed with certain inherent elementary properties, conditions or forces, necessary and essential for the production of vital phenomena.

2nd. That the elementary forces of all organized structures, are in no way derived from any connection with the nervous system, but are distinct, inherent and peculiar.

3rd. That the elementary properties and functions of the animal tissues are susceptible to various influences and changes through the medium and quality of the blood.

4th. That a first class of medicines, called stimulants, of which alcohol is the type, manifest a primary and specific action upon the brain and nerves, or nerve centres, which they influence. Their action is transitory, exalting nerve-force. That coincident with their action upon these structures, they exert a direct and deleterious influence upon the component elements of the blood and tissues, with the ultimate effect of lowering the elementary vital properties of the body—tonicity and susceptibility.

5th. That a second class of medicines, called tonics, act while in the blood, improving its quality and composition, and thereby the tonicity and firmness of the general system. That their action is slow and permanent—exalting vital force."

In illustrating the first three propositions, Dr. Parker enters upon a true physiological discussion concerning the elementary properties and functions of organized and living matter, and their capability of being acted on or modified by medicinal agents. This seemed to constitute a necessary introduction to the main subjects embraced in the fourth and fifth propositions. For it is evident that there can be no clear conception of the *modus operandi*, of a medical agent, without a previous definite knowledge of all those properties and functions of the living structures which are susceptible of being acted upon or modified.

To sustain his *fourth* proposition the author introduces several interesting experiments with alcoholic drinks, camphor and carbonate of ammonia, together with a summary of the opinions of Percy, Prout, Liebig, Carpenter, Pereira, and Ducheck.

From all his investigations, he educed the following general conclusions concerning the action of alcoholic stimulants, viz:

6th. That the popular belief, that the action of alcoholic stimulants is confined to the brain and nervous system alone, simply inducing mental exhileration, and a temporary exaltation of nervous power, is not true; but that coincident with their action upon these structures, they exert in a variable degree, a direct and deleterious influence upon the component elements of the blood and tissues, with the ultimate effect of lowering the elementary vital properties of the body, tonicity and susceptibility.

This they accomplish in the following manner:

1st. By their great affinity for, and absorption of the oxygen of the blood, thereby interfering with its agency in the formation of plastic material, and impairing the organizability of those compounds designed for nutrition and reproduction. 2nd. By preventing or retarding that vital change—the *conversion of venous blood into arterial*, and diminishing the functional activity of the secreting and excreting structures generally, thus causing a retention and accumulation in the blood of effete and excrementitious compounds. 3d. By retarding capillary circulation and the metamorphosis of the tissues.

7th. That alcoholic stimulants diminish animal heat, and the exhalation of carbonic acid.

8th. That the popular notiod that alcoholic stimulants promote healthy digestion and chymification, is not true, and therefore should be discountenanced and discouraged by medical men, not only as failing in this, but as destroying the natural sensibility of the stomach, and inducing morbid irritability and diseased action.

9th. That the stimulation and excitement which naturally follows the use of these substances, tends to destroy that harmony of action between the different organs and forces of the body, upin which their functional activity, and the continuance and maintenance of health depends. Finally,

"That alcoholic stimulants are the most unfit, and the least calculated of all other expedients, to impart strength and vitality to the duman system, or enable it to resist the depressing influences of cold or hunger, fatigue or disease, or any of the various circumstances and events to which it may be exposed."

To sustain his fifth proposition, the author introduces no new experiments, but relies on the general observations of writers concerning the action of tonics. He even omits, in this part of his essay, many interesting and valuable facts bearing upon the subject, which a more extended examination of our medical literature would have disclosed to his view. The general conclusions which he deduces from this part of his task are stated as follows:

1st. That tonics are hæmatic medicines, and as such, are restorative in their influences; that, unlike *neurotics* and *stimulant*, they have no direct or special action upon nerve matter.

2nd. That they are not foreign to the blood, and may therefore remain in it: that they improve its quality, and the plasticity of its organizable material, assisting digestion and promoting assimilation and nutrition. Hence they are slow, but permanent in their effects

3d. That they exalt the tone of the muscular fibre; and hence by their use, "the pulse becomes fuller, stronger and regular, and the muscular power increased,"—that they increase the cohesion and density of the tissues—diminish profuse secretions when dependent upon atony and debility, and finally, augment the vital forces of the system in general, thereby restoring *susceptibility* when defective, and the properties and functions of the nervous system to their normal condition.

Our design in noticing this essay, is simply to give the reader such an idea of its general scope and matter as will induce him to procure and read it for himself, rather than to examine it with the pen of a critic. Were we disposed to assume the latter position, however, we should be compelled to question the correctness of some parts of the *fourth* general proposition, already quoted. In it the author asserts that, "their action (alcoholic stimulants) is transitory, *exalting* nervous force." That this class of agents exert a primary influence on the brain, directly increasing nervous exhileration, excitement or susceptibility, we admit.

But the expression "*exalting nervous force*," plainly implies an increase of nervous energy, power, or strength; which is certainly not one of the effects of alcohol. Under its influence an individual may manifest rapidity of thought, social vivacity, and acuteness of perception, but we seek in vain for, even a temporary increase of true nervous energy or strength. Again the assertion that "they (stimulants) exert a direct and deleterious influence on the component elements of the blood and tissues," though true so far as regards alcohol, is by no means equally so in regard to all stimulants. Thus Gum Camphor, tea and coffee are all stimulants in the sense given to that term by our author. The first is included by him in the list of articles experimented with. But we can discover nothing in the results of his experiments, or in the observations of others which would countenance the idea that they exert any special deleterious influence, either on the elements of the blood or of the organized tissues of the body.

But these errors, (if errors they are), arise more from the want precision in the use of language than from erroneous ideas in the mind of the author.

The essay, as a whole, is one highly creditable to the author, and well worthy of careful perusal by the profession. There are one or two things, however, in the essay which require explanation. Readers of the Journal will recollect that nearly two years since I commenced publishing in its columns a series of articles on the "Pathology of Fevers." In one of the earlier numbers of this series, I detailed two experiments designed to show the influence of alcoholic drinks on the human system, and especially on the functions of Respiration and Calorification.

These experiments were dated, the first, Oct. 18, 1852, and the second, Nov. 8th, of the same year. They are there introduced as part of a series of experiments "I commenced in the winter of 1850," and they are represented throughout as having been conceived and executed by me.

The reader will readily perceive that the two first experiments detailed in the prize essay are the same as previously published by me. They are introduced by Dr. Parker as though they had been conceived and executed directly under his own supervision, simply acknowledging in a marginal note that he had been *assisted* by me. If he had said in the marginal note that they were part of a series which he had witnessed in my office, and which he had been authorized to use, it would have saved the necessity for this explanation. Beside this, however, justice required that he should have made some reference to one or more similar experiments instituted by me in the winter of 1850, and published by me in this Journal during the summer of 1851, after having been read to the American Medical Association, during its meeting in Charleston, S.C. We shall say nothing of the striking similarity between the views presented in the Essay concerning the *elementary properties* and functions of organized matter, and those previously published by me in the articles on the Pathology of Fevers. Indeed, we would have remained silent concerning the experiments also, had it not been for the fact, that they constitute part of a series on which we have bestowed much labor through several years, and which we intend at some future day to communicate to the profession more in detail.

Accompanying the address of Prof. Brainard on Poisoned Wounds, are two lithograph plates which enhance the value of the work, and with the quality of the matter contained, make the present volume of Transactions of our State Society equal in interest and importance to those published by other State organizations. D.

